# Review of Management and Outcomes in Women with Thrombophilia Risk during Pregnancy at a Single Institution

**DOI:** 10.1155/2014/381826

**Published:** 2014-02-17

**Authors:** Alhossain A. Khalafallah, Abdul-Rauf O. Ibraheem, Qiong Yue Teo, Abdul-Majeed AlBarzan, Ramanathan Parameswaran, Emily Hooper, Toly Pavlov, Amanda E. Dennis, Terry Hannan

**Affiliations:** ^1^Department of Clinical Haematology, Launceston General Hospital, Launceston, TAS 7250, Australia; ^2^Department of Medicine, Launceston General Hospital, Launceston, TAS 7250, Australia; ^3^School of Human Life Sciences, University of Tasmania, Launceston, TAS 7250, Australia; ^4^Menzies Research Institute, Hobart, TAS 7000, Australia; ^5^Department of Obstetrics and Gynaecology, Launceston General Hospital, Launceston, TAS 7250, Australia

## Abstract

Pregnancy is a hypercoagulable state associated with an increased risk of venous thromboembolic disease (VTE). We retrospectively studied 38 Caucasian pregnant women with thrombophilia risk and compared their obstetric outcomes with a matched cohort without known thrombophilia risk during the period between January 2007 and December 2010. There were (2) cases with factor V Leiden, (6) prothrombin gene mutation, (1) antithrombin III deficiency, (2) protein C deficiency, (3) protein S deficiency, (10) MTHFR mutation, (7) anti-cardiolipin antibodies, and (1) lupus anticoagulant. Patients without thrombophilia who presented with recurrent unprovoked VTE were considered as high risk (6 cases). Most patients received anticoagulation (34/38) with aspirin only (6), enoxaparin (27), and warfarin (1). Twenty-six out of thirty-eight pregnant women (68.4%) with an increased risk of thrombophilia experienced one or more obstetric complications defined as hypertension, preeclampsia, placenta abruptio, VTE, and oligohydramnios, compared with 15 out of 40 (37.5%) pregnant women in the control group (OR 3.6; 95% CI 1.42, 9.21, *P* < 0.001). The incidence of obstetric complications was significantly higher in the thrombophilia group compared to the controls. However, these complications were the lowest among patients who received full-dose anticoagulation. Our study suggests that strict application of anticoagulation therapy for thrombophilia of pregnancy is associated with an improved pregnancy outcome. The study was registered in the Australian and New Zealand Clinical Trials Registry under ACTRN12612001094864.

## 1. Introduction

Pregnancy is associated with major physiological changes that affect coagulation and the fibrinolytic system [1–3]. An imbalance in this system leads to a hypercoagulable state and pregnant women are therefore at an increased risk of venous thromboembolic disease (VTE), especially if they are affected by an associated acquired or inherited thrombophilia [2–4]. There are two factors that may exaggerate this risk: the high-risk nature of the thrombophilia and a history of a previous unprovoked VTE [5, 6]. High-risk hereditary thrombophilia includes antithrombin deficiency, prothrombin gene mutation (PGM), and factor V Leiden (FVL), while the presence of lupus anticoagulant or anti-cardiolipin antibodies are considered as acquired risk factors [7, 8]. Furthermore, homozygosity or presence of a combination of thrombophilia factors will aggravate the VTE risk by certain fold [7–9].

Apart from the occurrence of VTE, maternal thrombophilia has also been variably associated with an increased risk of early miscarriages, intrauterine growth restriction (IUGR), and pregnancy loss [10, 11].

Although it may seem intuitive to treat pregnant women with high-risk thrombophilia with anticoagulants prophylactically, there is a paucity of randomised trials in this area, and the balance of intervention versus conservative management should be carefully evaluated from both fetal and maternal points of view. Evidence-based guidelines have been published in an attempt to provide a more uniform clinical approach; however, there appears to be a lack of consistency among different guidelines [12, 13].

The decision to recommend anticoagulant prophylaxis to women with thrombophilia is based on the risk assessment or balance of bleeding versus VTE risk, as well as the potential effect that VTE and anticoagulants can have on pregnancy [14]. However, the use of anticoagulants in pregnancy is challenging because of the potential maternal and fetal complications [15, 16]. Despite this and the lack of controlled trials, there has been increased use of anticoagulants to prevent VTE and adverse pregnancy outcomes [17].

In this retrospective study, the management strategies of seventy-eight Caucasian women who received antenatal care at a single institution during the period between January 2007 and December 2010 were reviewed and analysed. Thirty-eight women with a thrombophilia risk and forty consecutive pregnant women who served as a control group received antenatal care at a single institution to determine the best management strategies based on the outcomes of the pregnancies.

## 2. Methods

### 2.1. Participants

The study was approved by the Human Research Ethics Committee. The study was registered in the Australian and New Zealand Clinical Trials Registry at http://www.anzctr.org.au/ under ACTRN12612001094864.

Thirty-eight pregnant women with a median age of 30 years and positive history for VTE or confirmed thrombophilia tests were recruited from the Queen Victoria Maternity Unit (QVMU) at our institution. All had been attending the antenatal clinic since the confirmation of a positive pregnancy test. At the same time, forty pregnant subjects with a median age of 27 years attending the QVMU antenatal clinic during the study period served as controls (Table 1). As per our standard practice, the patients with established history of thrombophilia were referred to a special clinic run by the Haematology Department at the LGH. All patients with thrombophilia risk who attended this high-risk clinic at our institution were included in this observational study. As a part of routine assessment of high-risk patients, a thrombophilia screen was performed for all cases.

The patients' medical records were reviewed by a trainee of the Royal Australian and New Zealand College of Obstetricians and Gynaecologists and supervised by senior obstetricians. All patients were co-managed by a haematologist who assessed the patients and determined the need for anticoagulation and the appropriate anticoagulant regimen as per the Royal College of Obstetricians and Gynaecologists (RCOG) 2004 guidelines “Thromboprophylaxis during Pregnancy, Labour and after Vaginal Delivery” and their subsequent edition in 2009 “Green-Top Guideline 37a” [12]. An assessment form was designed and used to collect medical, obstetric, and family history for each patient as well as risk factors for thrombosis and previous VTE. Outcomes of previous and current pregnancies were also recorded.

### 2.2. Determination of Thrombophilia Risk

All blood tests were performed during the first antenatal assessment and included assays of antithrombin III, protein C activity, free and total antigen protein S, activated protein C resistance, anti-cardiolipin antibodies (IgG and IgM), lupus anticoagulant, and fasting plasma homocysteine. Genetic studies were performed to look for factor V Leiden mutation if the patients had an elevated activated protein C resistance (APCR), prothrombin gene mutation, and homozygous status for the gene encoding methyltetrahydrofolate reductase enzyme (MTHFR). Other risks of thrombophilia including immobilisation, the presence of recent major surgery, trauma, malignancy, and family history of VTE were obtained from the patients. The prevalence of the various risk factors in the group of pregnant women with suspected or confirmed thrombophilia is shown in Table 2. Data of age and parity matched, as possible, control pregnant women were collected from 40 consecutive pregnancies at the same institution without known thrombophilia risks and history suggestive for VTE and served as a control group. No anticoagulation treatment was initiated in the control group.

Criteria of diagnosis of DVT were based on ultrasound Doppler in these cases. A follow-up ultrasound was instated to check for residual thrombosis in positive cases.

The British RCOG Guideline for risk stratification was applied (Green-Top Guideline 37a 2009) [12]. Low-risk patients received treatment with oral aspirin 100 mg daily only throughout pregnancy (6 cases) versus no treatment (4 cases). Intermediate risk patients received prophylactic treatment with enoxaparin 40 mg subcutaneously daily throughout pregnancy (11 cases) in combination with aspirin 100 mg orally daily in case of the presence of antiphospholipid syndrome, lupus anticoagulant, or SLE (6 cases). High-risk patients (11 cases) received treatment doses of low molecular weight heparin (LMWH) with enoxaparin as 1 mg/Kg body weight subcutaneously twice daily or 1.5 mg/Kg body weight subcutaneously daily. One patient was on oral vitamin K antagonist (warfarin) therapy prior to her unplanned pregnancy and then switched to LMWH once the pregnancy was confirmed at 8 weeks. Folic acid 5 mg taken orally daily was added in case of homozygous status of MTHFR. Anticoagulation was continued for at least 4 weeks postpartum in the intermediate and high-risk groups.

### 2.3. Statistical Analysis

A multivariate analysis of pregnancy outcome in correlation with risk factors of pregnancy was performed by using Stata 10.0 software. Numerical data was summarised with means and standard deviations, categorical data with numbers and percentages. Adjustment for putative confounders such as age, parity, and other risk factors was performed with unconditional logistic regression. Relative risks associated with laboratory thrombophilia abnormalities were expressed as odds ratios, with 95% confidence intervals. The association between the categorical variables was assessed by Fisher's exact test for small groups. For noncategorical variables, Student's *t* test was used. Furthermore, *P* value < 0.05 was considered as statistically significant.

## 3. Results 

**Figure 1:**
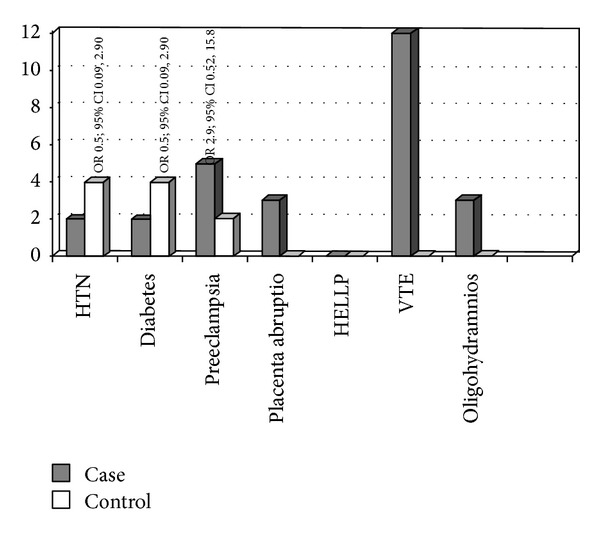
Prevalence of obstetric complications in both cases and controls. Complications of pregnancy referred to HTN, hypertension; diabetes; preeclampsia; placenta abruptio; IGUR, intrauterine growth restriction; IUFD, intrauterine fetal death; VTE, venous thromboembolic disease; oligohydramnios; stillbirth.

Twenty-six out of thirty-eight pregnant women (68.4%) with increased thrombophilia risk experienced one or more obstetric complications defined as diabetes, hypertension, pre-eclampsia, placenta abruptio, VTE, or oligohydramnios, compared with 15 out of 40 (37.5%) pregnant women in the control group (OR 3.6; 95% CI 1.42, 9.21, *P* < 0.001). The prevalence of obstetric complications in both thrombophilia cases and the controls is given in Figure 1. VTE appears to be the most common obstetric complication experienced by the group of pregnant women with an increased thrombophilia risk (12/38) and occurred during the first trimester as proximal DVT. Of the thirty-eight pregnant women in the thrombophilia risk group, only four did not commence anticoagulant treatment because of relatively low risk such as presence of heterozygous status of MTHFR gene mutation with family history of VTE without additional risk factors. Some guidelines consider that MTHFR is not a thrombophilia risk factor; however we included only the homozygous cases.

Six out of ten women, who received anticoagulation therapy before pregnancy because of recurrent or recent VTE and continued during pregnancy, experienced obstetric complications compared to 12 out of 18 women who were offered anticoagulants after confirmation of pregnancy in the antenatal period. The incidence of obstetric complications such as pre-eclampsia, placenta abruptio, oligohydramnios, and VTE was significantly higher in the women of the increased thrombophilia risk group compared to the control group (*P* < 0.001) (Figure 1).

Types of anticoagulant used during pregnancy are demonstrated in Table 3 and obstetric complications observed are given in Table 4. The pregnant women with thrombophilia risk classified as high risk (11/38), moderate risk (17/38), or low risk (10/38) were noted to have a similar rate of obstetric complications (Table 4). In the high-risk group (11 cases) all women received full doses of anticoagulant during the prenatal and/or antenatal period and 5 of 11 women were observed not to develop any complications. In the moderate-risk group (17 cases), 2 women did not use anticoagulant and 1 of the 2 women who did not use anticoagulant experienced obstetric complications. Interestingly the low-risk group (10) was observed to have a higher rate of obstetric complications (70%) as compared to the high (54%) and intermediate (59%) groups. Of the low-risk group, 4 out of 5 women who were not anticoagulated did experience obstetric complications such as hypertension, pre-eclampsia, placenta abruption, and oligohydramnios. There was no incidence of major or serious bleeding in our cohort of patients who received anticoagulation. There was an association between maternal weight and occurrence of VTE during pregnancy, but as yet not statistically significant (Table 1(b)). The presence of inherited thrombophilia risk was not associated with VTE due to the fact that the patients were already fully anticoagulated. However previous history of VTE was associated with significant risk of developing recurrence of VTE in pregnancy (*P* = 0.02). Furthermore there was no statistical difference between the thrombophilia group and the control group in terms of the incidence of IUGR, IUFD, small for gestational age (SGA), and stillbirth.

## 4. Discussion

Risk factors for VTE in pregnancy include age over 35 years, obesity, positive thrombophilia screen, gross varicose veins, immobility including long distance travel, dehydration, infective and inflammatory conditions such as inflammatory bowel disease and pre-eclampsia, major obstetric haemorrhage, medical conditions such as nephrotic syndrome, and operative delivery, especially emergency caesarean sections in labour [18]. A significant added risk to thrombosis is the presence of coexistent inherited thrombophilia during pregnancy [19]. The presence of genetic thrombophilia markers such as FVL, PGM 20210A mutation, AT III deficiency, and antiphospholipid antibodies significantly increases a patient's risk of thrombotic event [20, 21]. Thromboembolic events are reported in approximately one-third of antiphospholipid-positive patients [22]. As thrombotic events during pregnancy are frequently seen in those with thrombophilia defects [8], it emphasizes the importance of taking a careful obstetric history in all patients as part of their risk assessment profile. Pre-eclampsia, stillbirth, placental insufficiency and the haemolysis, elevated liver enzymes, and low platelet count (HELLP) syndrome are obstetric complications that may occur in thrombotic events in pregnancy [10]. However, there was no statistical difference between the studied two groups regarding the incidence of IUGR, IUFD, SGA, and stillbirth. This could be due to the small number of cases. Moreover, constitutional factors are known to account for up to 70% of SGA [23]. The most likely explanation of the low-risk group having a higher rate of obstetric complications compared to other risk groups is the use of full anticoagulation therapy in the high-risk groups. Thus the pregnancies were associated with a lower incidence of obstetric complications in these groups albeit the high thrombophilia risk. However this issue should be addressed separately in specially designed trials, as there are many variables and due to the relatively small number in our cohort of patients.

Although previous reports suggested that the highest-risk period for VTE is the late third trimester and postpartum period, our study has shown that the antepartum period has the highest risk for deep venous thrombosis (DVT), with 12/38 cases of DVT occurring in the first trimester [24]. Refuerzo and colleagues concluded in the study of VTE in pregnancy that lack of definitive signs and symptoms of thromboembolic disease during pregnancy warrants a complete evaluation of patients clinically suspected of having VTE [25]. There is a lack of trials that assess the safety and accuracy of objective testing in pregnancy and no evidence to suggest that routine screening in pregnancy is cost-effective in low-risk populations [26]. VTE remains a substantial problem despite the dramatic decline in pregnancy-related mortality in industrialized countries over the past century. Nevertheless, VTE is the main direct cause of maternal mortality and a major contributor to morbidity in pregnancy [27].

## 5. Conclusion

Thromboembolic disease is one of the major and increasing causes of morbidity and mortality in the developed world. Management of women with an increased risk of thrombophilia with active anticoagulation is associated with less complications and improved obstetric outcomes. Therefore, it is important to follow the available guidelines and recommendations such as those of the Royal College of Obstetricians and Gynaecologists [12], whenever possible, to achieve the best possible outcome in pregnancy. Further studies to confirm our findings are warranted.

## Figures and Tables

**Table:** (a)

Characteristic	Pregnant women with positive thrombophilic screening test (*n* = 38)	Pregnant women without positive thrombophilic screening test (*n* = 40)	*P* value
Mean age (SD)	30.58 (5.07)	27.38 (7.31)	
BMI (SD)	27.91 (5.08)	27.44 (7.30)	0.34
Primiparity	4	13	
Infant birth weight (g)^+^	3298	3249.23	0.79

**Table:** (b)

	OR	95% CI	*P* value
Maternal weight	23.2	(0.94 to 2,926)	0.067
History of thromboembolic events	9.52	(1.02 to ∞)	0.024
Inherited thrombophilia	1.00	(0.00 to 39.0)	1.00

**Table 2:** Prevalence of thrombophilia in the case population.

Thrombophilia risk	Number of cases
Inherited or acquired	
Factor V Leiden mutation	2
MTHFR mutation	10
Prothrombin gene mutation	6
Hyperhomocysteinemia	1
Protein C deficiency	3
Protein S deficiency	2
Antithrombin deficiency	1
Antiphospholipid syndrome	7
Lupus anticoagulant	1

Personal history of venous thrombotic event (VTE)	16
Positive family history of VTE	6

**Table 3:** Type of anticoagulant used in pregnancy.

Types of anticoagulant	Number of cases
Aspirin	6
Warfarin	1
Enoxaparin	18
Folic acid	1
Enoxaparin + aspirin	8
Enoxaparin + folic acid	1

**Table 4:** Risk classification and obstetric complications*.

Risk classification	Number of cases	% Obstetric complications (*n*)
Low	10	70% (7)
Moderate	17	59% (10)
High	11	54% (6)

*Obstetric complications defined as hypertension, pre-eclampsia, placenta abruptio, VTE, oligohydramnios, IGUR, IUFD, and stillbirth.

## Abbreviations

APCR: Activated protein C resistance
DVT: Deep venous thrombosis
FVL: Factor V Leiden
HELLP: Haemolysis, elevated liver enzymes, and low platelet count syndrome
IUFD: Intrauterine fetal death
IUGR: Intrauterine growth restriction
LMWH: Low molecular weight heparin
MTHFR: Methyltetrahydrofolate reductase
PGM: Prothrombin gene mutation
VTE: Venous thromboembolic disease.
